# Universal expression for the drag on a fluid sphere

**DOI:** 10.1371/journal.pone.0194907

**Published:** 2018-04-16

**Authors:** D. A. Barry, J.-Y. Parlange

**Affiliations:** 1 Ecological Engineering Laboratory (ECOL), Environmental Engineering Institute (IIE), Faculty of Architecture, Civil and Environmental Engineering (ENAC), Ecole polytechnique fédérale de Lausanne (EPFL), Lausanne, Switzerland; 2 Department of Biological and Environmental Engineering, Cornell University, Ithaca, New York, United States of America; Huazhong University of Science and Technology, CHINA

## Abstract

An expression was developed for prediction of drag coefficients for any spherical particle, drop or bubble in an infinite, homogeneous liquid. The formula reproduces the limiting cases for gas bubbles and solid spheres, as well as the exact Hadamard-Rybczynski solution. The accuracy of the expression, which is valid for Reynolds numbers up to a few hundred, was confirmed by comparison with published numerical predictions of the drag coefficient for a range of physical circumstances.

## Introduction

Bubbles, drops and particles are widespread in science and engineering phenomena. Knowledge of the behavior of single bubbles and drops is not only directly relevant to many applications; it also supports understanding of the corresponding swarms, e.g., [1].

Wegener et al. [2] recently provided a comprehensive review of theory, experimental data and pertinent approximations describing the dynamics of single drops in fluid systems. The steady rate of movement of spherical particle, drops and bubbles is quantified by the drag coefficient, *C*_*d*_. Of relevance here is Wegener et al.’s summary of drag formulas (their Tables 1 and 2), and the ranges of (drop) Reynolds numbers over which the different formulas apply.

We consider the drag on a single spherical solid particle, liquid drop or gas bubble (collectively referred to as a particle) moving in an otherwise quiescent, infinite, homogeneous liquid, without interphase mass transfer (i.e., Sherwood number of zero). Spherical particles will occur if surface tension is dominant, or if inertia is negligible. Clift et al. [3] and Bhaga and Weber [4] provide systematic characterizations of particle shape as it varies with Reynolds, Eötvös and Morton numbers. These authors show, for instance, that spherical shapes for Reynolds numbers of about 100 or higher are found for sufficiently small Eötvös and Morton numbers.

Below, we develop and test a new expression for *C*_*d*_ applicable to spherical particles. Our approach is to build an interpolation based on known limiting cases (e.g., small Reynolds number), as well as a validated large Reynolds number drag expression.

## Theory

### Drag formula interpolations between gas bubbles and solid spheres

There are several approximations for *C*_*d*_ in the literature for the limiting cases of bubbles or solid spheres [5–8]. As mentioned, Wegener et al. [2] provide a summary of these approximations, remarking that they “approximate only certain intervals of the standard drag curve.”

A well-known interpolation is that of Rivkind and Ryskin [9]:

$$C_{d}≅\frac{X\left(24R_{0}^{-1}+4R_{0}^{-1/3}\right)+14.9R_{0}^{-0.78}}{1+X},$$ (1)

with

$$R_{0}=2U\rho_{0}a\mu_{{}_{0}}^{-1};X=\mu_{1}\mu_{{}_{0}}^{-1},$$ (2)

where *ρ* is the density, *μ* the viscosity, *U* the (steady) velocity of the sphere, *R*_0_ the Reynolds number, *X* the viscosity ratio (*X* → 0 for a gas bubble and ∞ for a solid sphere) and *a* the sphere radius. Subscript “0” refers to the material outside the sphere and “1” to that within. Although Eq (1) is straightforward, it does not approach known limits, as given below.

### Analytical drag results for small Reynolds number

For *R*_0_ → 0, the exact Hadamard-Rybczynski limit, e.g., [3], valid for all *X*, is:

$$C_{d}(R_{0}\ll 1)=\frac{16}{R_{0}\left(1+X\right)}\left(1+\frac{3}{2}X\right).$$ (3)

For the solid sphere, the small-*R*_0_ limit is [10]:

$$C_{d}(X\to \infty ,\;R_{0}\ll 1)=\frac{24}{R}+\frac{9}{2}+O(R_{0}\;\ln\;R_{0}).$$ (4)

Oliver and Chung [11] recommended using Eq (1) for 2<*R*_0_≤50 and, for *R*_0_≤2:

$$C_{d}(R_{0}\leq 2)≅\frac{16}{R_{0}\left(1+X\right)}\left(1+\frac{3}{2}X\right)+\frac{\chi }{{\left(1+X\right)}^{2}}{\left(1+\frac{3}{2}X\right)}^{2}.$$ (5)

with *χ* = 8/5 [12]. If we instead take *χ* = 2 in Eq (5), then it gives the exact small-*R*_0_ result for a solid sphere, Eq (4)

### Analytical drag results for large Reynolds number

Unlike the situation for *R*_0_ ≪ 1, exact results for *R*_0_ ≫ 1 do not exist. For laminar flow (i.e., oscillations of the particle do not occur), Harper and Moore [13] as well as Parlange [14] obtained approximate expressions for the drag for this case, however. In both [13] and [14], it was observed that, to a first approximation, flow inside the particle is described by a Hill’s vortex, and outside by a potential flow. Both approaches give drag predictions that are “numerically indistinguishable” [14]. This conclusion follows from the minor effect on the drag made by slightly different assumptions in [13] and [14]. Barry and Parlange [15] compared predictions of both theories to experimental results on recirculation within the particle [4], and found that the theory of Parlange [14] is more accurate. Thus, this theory is the starting point for the developments presented below.

The drag formula of Parlange [14] is:

$$\begin{array}{c} C_{d}=\frac{48}{R_{0}}\left(1+\frac{3}{2}X\right)\left[1-\frac{4}{5}{\left(\frac{2}{\pi R_{0}}\right)}^{1/2}\left(1+\frac{3}{2}X\right)\left(6\sqrt{3}+5\sqrt{2}-14\right)Z\right]\;\text{with}\\ Z=1+\left[\left(\alpha -2\right){\left(XP\right)}^{1/2}+\left(\beta -1\right)XP\right]{\left[1+{\left(XP\right)}^{1/2}\right]}^{-2}, \end{array}$$ (6)

where *P* = *ρ*_1_$\rho_{0}^{-1}$ is the density ratio. The two constants, *α* and *β*, are defined by integrals. Parlange [14] simplified *Z* by taking *α* = 2 and *β* = 1. Numerical evaluations (Appendix) give *α* and *β* as:

α=2.5891,β=0.9879. (7)

In the following, we use *α* and *β* as given by Eq (7) For later convenience in manipulating Eq (6), we define *A* as:

$$A=\frac{2}{5}\sqrt{\frac{2}{\pi }}\left(6\sqrt{3}+5\sqrt{2}-14\right)≅1.10535.$$ (8)

### New drag formula

Eq (6) holds for *R*_0_ ≫ 1, so it is not surprising that Eq (3) (the Hadamard-Rybczynski limit) is not obtained for *R*_0_ ≪ 1. However, we can force Eq (6) to do so by using standard Padé approximations [16]. First, we rewrite Eq (6) as:

$$C_{d}=\frac{16}{R_{0}\left(1+X\right)}\left(1+\frac{3}{2}X\right)\left(R_{0}^{1/2}+AZ\right){\left[\frac{R_{0}^{1/2}}{3}{\left(1+X\right)}^{-1}+AZ\right]}^{-1},$$ (9)

which is identical in order to Eq (6) but additionally approaches Eq (3) as *R*_0_ → 0. Next, we modify Eq (9) so that it will reduce to Eq (5) (with *χ* = 2), in which case it must be corrected for *R*_0_ ≪ 1 without affecting predictions for *R*_0_ ≫ 1. This occurs with a Padé approximant that maintains the first two orders of Eqs (4) and (6) for *R*_0_ small and large, respectively. We proceed stepwise and satisfy the different limiting cases given above. First, to ensure that the predictions of Eq (9) are not affected for *R*_0_ ≫ 1, we take corrections that are exponentially small in that limit, and so replace Eq (9) with:

$$C_{d}=\frac{48}{R_{0}\left(1+X\right)}\left(1+\frac{3}{2}X\right)\frac{R_{0}^{1/2}+AZ+\tau \exp\left(-\lambda R_{0}^{1/2}\right)}{R_{0}^{1/2}{\left(1+X\right)}^{-1}+3AZ+3\tau \exp\left(-\omega R_{0}^{1/2}\right)},$$ (10)

which reduces to Eq (9) for *R*_0_ ≫ 1 (or for *τ* = 0) and Eq (3) for *R*_0_ → 0. Second, the parameters *τ*, *λ* and *ω* (all > 0) are chosen to ensure that Eq (10) reduces to the other limits given above. For this purpose, we observe that Eq (5) requires a Padé approximant in powers of *R*_0_, rather than $R_{0}^{1/2}$, appearing in Eq (10) The initial appearance of $R_{0}^{1/2}$ is removed from the small-*R*_0_ expansion of Eq (10) if:

$$\lambda \tau =1\;and\;\omega \tau =\frac{1}{3\left(1+X\right)}.$$ (11)

In addition, to satisfy Eq (5) (with *χ* = 2) for *R*_0_ ≪ 1 requires that *τ* is given by:

$$\tau \left(\tau +AZ\right)=\frac{8}{9}\left(\frac{4+3X}{1+X}\right).$$ (12)

Once *τ* is obtained using Eq (12), *λ* and *ω* follow straightforwardly from Eq (11).

The only arbitrary element in the deviation of Eq (10) is the form of the exponential corrections. Alternative functional forms were investigated, with the most promising being $\beta {\left(1+\lambda R_{0}^{1/2}\right)}^{-1}$. However, based on comparisons with published numerical results (below), we found that only the exponential form reduced to Eq (9) rapidly enough for *R*_0_ ≫ 1.

Eq (10) constitutes a new, fully analytical, expression for the drag of a spherical particle. It reduces quickly to Eq (9) for *R*_0_ ≫ 1. All coefficients are determined by the behavior of *C*_*d*_ under different conditions, i.e., no empirical coefficient is determined by curve fitting of numerical results.

Because the bubble (*X* = 0) and the solid sphere (*X* → ∞) are oft-investigated special cases, we present Eq (10) for these limits.

### Bubble (*X* = 0)

$$C_{d}=\frac{48}{R_{0}}\frac{\sqrt{R_{0}}+A+\tau_{X=0}\exp\left(-\sqrt{R_{0}}/\tau_{X=0}\right)}{\sqrt{R_{0}}+3A+3\tau_{X=0}\exp\left(-\sqrt{R_{0}}/3\tau_{X=0}\right)};2\tau_{X=0}=\sqrt{A^{2}+\frac{128}{9}}-A.$$ (13)

### Solid sphere (*X* → ∞)

$$C_{d}=\frac{24}{R_{0}}\frac{\sqrt{R_{0}}+A\beta +\tau_{X\to \infty }\exp\left(-\sqrt{R_{0}}/\tau_{X\to \infty }\right)}{A\beta +\tau_{X\to \infty }};2\tau_{X\to \infty }=\sqrt{A^{2}\beta^{2}+\frac{32}{3}}-A\beta .$$ (14)

### Comparison with numerical results

Eq (10) is compared with numerical results from the literature. For convenience, numerous comparisons are collected in S1 Text. Specifically, tables of results from Eq (10) are compared with those from [3,11,17–27]. Representative results are presented below.

**Fig 1:**
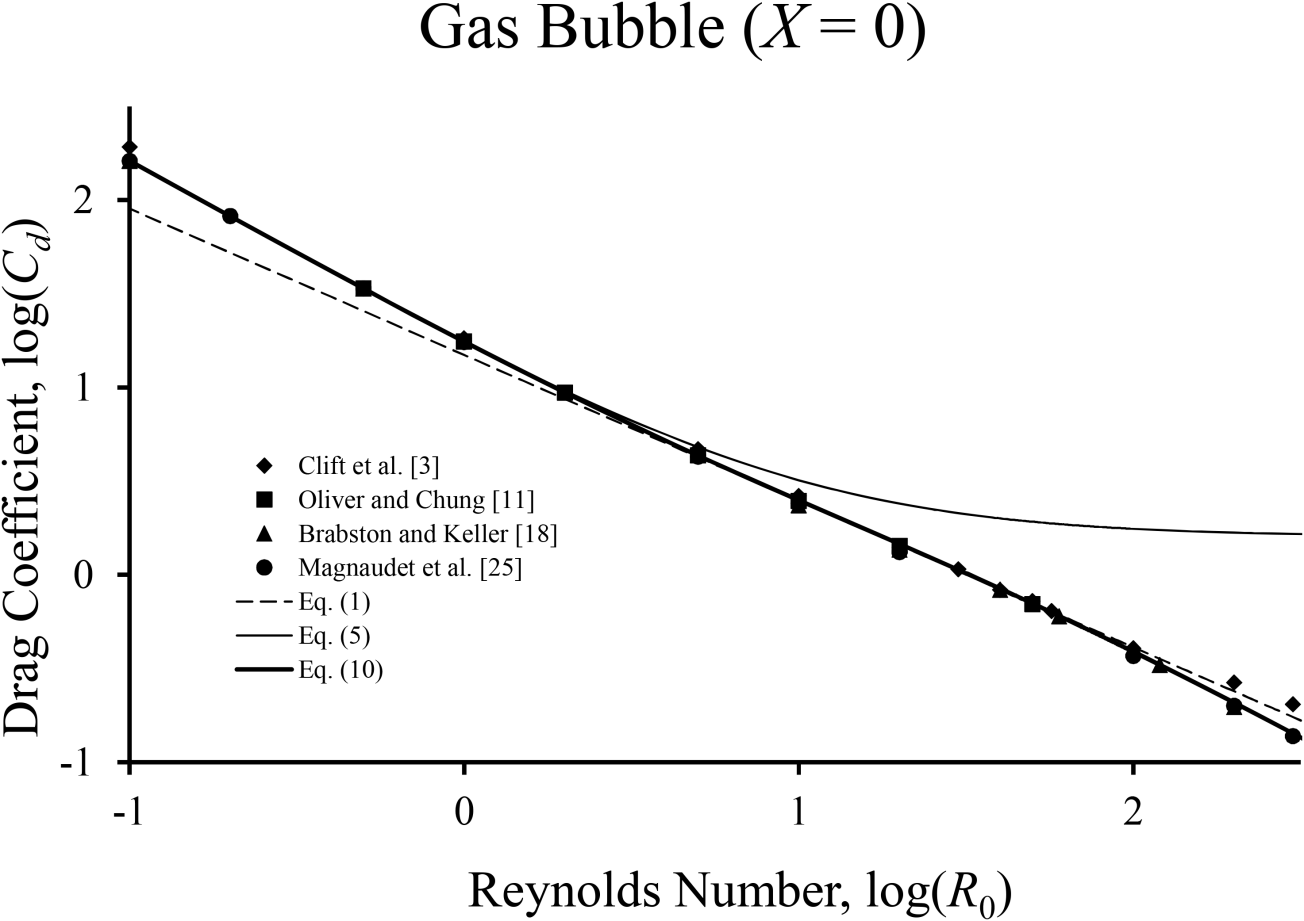
Comparison of various numerical data sets with predictions of drag formulas for the case of the gas bubble.

First, we consider in Fig 1 the gas bubble (*X* = 0), where results from the literature are collected. The figure compares Eq (10) with values given by Clift et al. [3], Oliver and Chung [11], Brabston and Keller [18] and Magnaudet et al. [25]. For the data of Clift et al. [3], the predictions agree well with the published values except for *R*_0_ ≥ 200. This is not surprising as Clift et al. [3] obtained values at *R*_0_ = 300 and 400 by interpolation with higher Reynolds numbers, when Eq (10) does not apply. Eqs (1) and (5) are accurate over their reported ranges of validity. The numerical simulations of Brabston and Keller [18] for *R*_0_ up to 200 are in close agreement with Eq (10). The accuracy of the numerical results of Brabston and Keller is confirmed from their agreement with the numerical results of Oliver and Chung [11] over the narrower range of 1/2 < *R*_0_ ≤ 50. The results of Magnaudet et al. [25] agree well with Eq (10) for the entire range of *R*_0_ considered.

**Fig 2:**
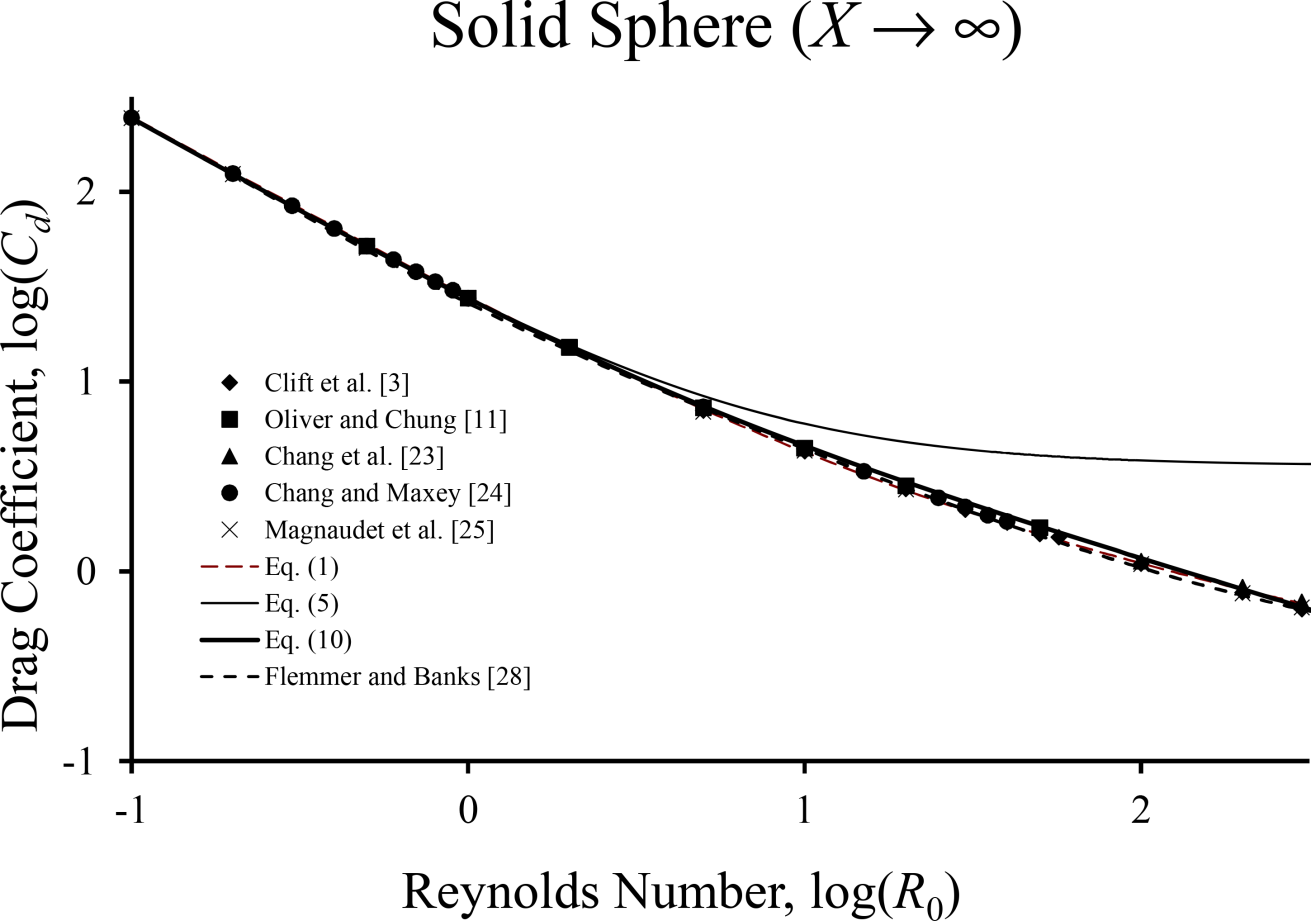
Comparison of various numerical data sets with predictions of drag formulas for the case of the solid sphere.

Fig 2 makes the same comparison for a solid sphere. Interestingly, the disagreement with the Clift et al. [3] values, and the predictions of the Rivkind and Ryskin [9] formula, Eq (1), is very small and is limited to *R*_0_ between approximately 10 and 100. Again, the drag values reported by Oliver and Chung [11], Chang et al. [23] and Chang and Maxey [24] are all similar, and agree more closely with Eq (10) than the results reported by Clift et al., although the results from Eq (10) seem slightly high. This figure includes results calculated from the formula of Flemmer and Banks ([28], Eq (7), which tends to be slightly lower than the numerical results. Besides the Flemmer and Banks [28] formula, there are several expressions for the solid sphere drag coefficient available. However, as shown by Mikhailov and Silva Freire [29], the largest variations between them occur at about *R*_0_ = 100, with a maximum deviation of about 5%, so the Flemmer and Banks formula can be taken as representative.

Comparisons for various *X* are given in Table 1, which lists drag values computed by Oliver and Chung [11] for *X* = 0, 0.333, 1, 3, **∞** and 1/2 ≤ *R*_0_ ≤ 50. The results for *X* = 0 and *X →* **∞** were already presented in the figures, of course. As expected, the agreement for intermediate *X* values is excellent and is similar to that shown in Figs 1 and 2. Table 1 includes the estimates calculated with Eq (1), which are less accurate than those from Eq (10).

**Table 1:** Drag coefficient, *C*_*d*_, estimated by Oliver and Chung [11] for various viscosity ratios (*X*) over a range of Reynolds numbers (*R*_0_) compared with predictions of Eqs (1) and (10).

*R*_0_ ↓	*X* →	0	0.333	1	3	∞
0.5	O&C^†^	33.8	38.2	42.7	47.2	51.8
	Eq (1)	25.6	32.4	39.3	46.2	53.0
	Eq (10)	33.7	38.1	42.6	47.1	51.7
1	O&C	17.6	20.0	22.5	25.0	27.5
	Eq (1)	14.9	18.2	21.5	24.7	28.0
	Eq (10)	17.5	19.9	22.4	24.9	27.5
2	O&C	9.4	10.8	12.2	13.6	15.1
	Eq (1)	8.68	10.3	11.9	13.6	15.2
	Eq (10)	9.39	10.8	12.2	13.6	15.1
5	O&C	4.33	5.02	5.75	6.50	7.28
	Eq (1)	4.25	4.97	5.69	6.42	7.14
	Eq (10)	4.33	5.04	5.80	6.59	7.42
10	O&C	2.48	2.94	3.43	3.93	4.45
	Eq (1)	2.47	2.92	3.37	3.81	4.26
	Eq (10)	2.50	2.96	3.48	4.02	4.59
20	O&C	1.43	1.74	2.09	2.45	2.81
	Eq (1)	1.44	1.75	2.06	2.37	2.67
	Eq (10)	1.46	1.78	2.14	2.55	2.96
50	O&C	0.70	0.90	1.15	1.44	1.72
	Eq (1)	0.705	0.920	1.14	1.35	1.56
	Eq (10)	0.703	0.896	1.14	1.44	1.73

^†^ Oliver and Chung [11]

Table 2 lists numerical results from Juncu [30], who considered different density ratios, *P*, for *R*_0_ = 100. We mention, in passing, that *C*_*d*_ is largely insensitive to changes in *P*, and if *P* is not specified, typically *P = X* is assumed (as done here). The agreement between the numerical results and Eq (10) is excellent. Again, the estimates of Eq (10) are usually slightly above the numerical values. Table 2 includes predictions from Eq (1), although this expression does not account for variations of *C*_*d*_ with *P*.

**Table 2:** Drag coefficient, *C*_*d*_, for *R*_0_ = 100 for different viscosity (*X*) and density (*P*) ratios. Roman values (dashes: No values provided) from Juncu [30], results from Eq (10) are in italics, results in the rightmost column are from Eq (1).

*X* *P* → ↓	0.01	0.1	0.2	0.5	1.0	2.0	5.0	10.0	100.0	Eq (1)
0.01	0.384	-	-	-	-	-	-	-	-	0.417
	*0*.*393*	*0*.*392*	*0*.*392*	*0*.*392*	*0*.*391*	*0*.*390*	*0*.*389*	*0*.*388*	*0*.*387*	
0.1	-	0.421	0.421	0.422	0.423	0.425	0.427	0.430	-	0.473
	*0*.*432*	*0*.*430*	*0*.*429*	*0*.*428*	*0*.*427*	*0*.*426*	*0*.*425*	*0*.*425*	*0*.*427*	
0.2	-	0.461	0.461	0.462	0.462	0.464	0.464	0.466	-	0.526
	*0*.*472*	*0*.*470*	*0*.*468*	*0*.*467*	*0*.*466*	*0*.*465*	*0*.*465*	*0*.*465*	*0*.*469*	
0.5	-	0.558	0.558	0.558	0.559	0.560	0.562	0.566	-	0.641
	*0*.*576*	*0*.*571*	*0*.*570*	*0*.*568*	*0*.*567*	*0*.*566*	*0*.*567*	*0*.*568*	*0*.*574*	
1.0	-	0.676	0.676	0.676	0.674	0.670	0.667	0.665	-	0.756
	*0*.*704*	*0*.*697*	*0*.*694*	*0*.*692*	*0*.*692*	*0*.*693*	*0*.*695*	*0*.*697*	*0*.*705*	
2.0	-	0.812	0.812	0.812	0.811	0.810	0.807	0.799	-	0.871
	*0*.*856*	*0*.*846*	*0*.*844*	*0*.*843*	*0*.*843*	*0*.*846*	*0*.*850*	*0*.*853*	*0*.*862*	
3.0	-	0.883	0.883	0.883	0.882	0.881	0.875	0.867	-	0.929
	*0*.*937*	*0*.*926*	*0*.*925*	*0*.*925*	*0*.*927*	*0*.*930*	*0*.*935*	*0*.*939*	*0*.*948*	
5.0	-	0.951	0.951	0.951	0.951	0.951	0.948	0.944	-	0.987
	*1*.*017*	*1*.*006*	*1*.*005*	*1*.*007*	*1*.*011*	*1*.*015*	*1*.*020*	*1*.*024*	*1*.*033*	
10.0	-	1.008	1.008	1.008	1.008	1.008	1.008	1.007	-	1.039
	*1*.*082*	*1*.*073*	*1*.*074*	*1*.*079*	*1*.*083*	*1*.*088*	*1*.*094*	*1*.*098*	*1*.*105*	
100.0	-	-	-	-	-	-	-	-	1.06	1.095
	*1*.*129*	*1*.*140*	*1*.*145*	*1*.*151*	*1*.*155*	*1*.*159*	*1*.*162*	*1*.*164*	*1*.*167*	

## Conclusion

We developed a formula, Eq (10), to predict the drag for a spherical particle, for all viscosity ratios between gas bubbles and solid spheres. It was derived as a Padé approximant that interpolates between known analytical results at low and moderate Reynolds numbers assuming that the particle does not oscillate or wobble. The formula can be used to predict the drag coefficient for spherical bubbles, drops and particles for any viscosity and density ratios. Surface tension is assumed sufficient to maintain the spherical shape of the particle. Both Fig 2.5 of Clift et al. [3] and Fig [8] of Bhaga and Weber [4] show how the particle shape changes (spherical, ellipsoidal, spherical cap, etc.) with the Eötvös, Morton and Reynolds numbers, and thus indicate the range of conditions for which Eq (10) applies. Eq (10) is more theoretically based than, for instance, the formula of Rivkind and Ryskin [9] and appears to be more accurate as well, especially at low Reynolds numbers.

## Appendix: Computation of *α* and *β*, Eq (7)

The definitions of *α* and *β*, which appear in Eq (6), are, respectively:

$$\begin{array}{c} 2\pi^{1/2}A\alpha =\left[\left(\sqrt{3}-\frac{2}{3}\right)\frac{24}{5}\sqrt{2}+\frac{8}{\sqrt{3}}\right]-\sqrt{\frac{2}{\pi }}\int_{0}^{\infty }\int_{-1}^{1}{\left(\frac{\partial N}{\partial Y}\right)}^{2}dYdr\\ -9\sqrt{3}\left[\frac{16}{7\pi }\left(\sqrt{3}-\frac{4}{3}\right)-\frac{1}{\sqrt{2\pi }}\int_{0}^{\infty }N\left(\frac{8}{9},Y\right)\text{ierfc}\left(\frac{3Y}{4\sqrt{2}}\right)dY\right], \end{array}$$ (A1)

$$2\pi^{1/2}A\beta =\frac{8}{\sqrt{3}}-\sqrt{\frac{2}{\pi }}\left[{\int_{0}^{\infty }\int_{-1}^{1}\left(\frac{\partial N}{\partial Y}\right)}^{2}dYdr+\frac{27}{16}\int_{0}^{\infty }N^{2}\left(\frac{8}{9},Y\right)dY\right].$$ (A2)

where ierfc is the integral of the coerror function [31]. Eqs (A1) and (A2) are obtained from the general definition of *C*_*d*_ ([13], Eq (8).1), with the velocity field of Parlange ([14], [18], [19] and [25]). *N* is defined as:

$$N\left(W,Y\right)=\frac{Y}{2\sqrt{\pi }}\int_{0}^{W}{\left(W-\bar{W}\right)}^{-3/2}\sin^{2}\theta \left(\bar{W}\right)\exp\left[-\frac{Y^{2}}{4}{\left(W-\bar{W}\right)}^{-1}\right]d\bar{W},0\leq W\leq Y,$$ (A3)

with ${\text{sin}}^{2}\theta (W)=1-4{\text{cos}}^{2}[\arccos\left(9W/4-1\right)/3+\pi /3]$, and where *θ*, *W* and *r* are related by:

$$r=\cos\left(\theta \right)\;\text{and}\;W=\frac{2}{9}{\left(1-r\right)}^{2}\left(2+r\right).$$ (A4)

The integrals appearing in Eqs (A1) and (A2) contain integrands that vary rapidly, viz., those containing *N*^2^ and (∂*N*/∂*Y*)^2^. However, the integral containing *N* and ierfc varies smoothly, and integrates to:

$$\frac{1}{\sqrt{\pi }}\int_{0}^{\infty }N\left(\frac{8}{9},Y\right)\;\text{ierfc}\left(\frac{3Y}{4\sqrt{2}}\right)dY≃0.29156.$$ (A5)

The integrands containing of *N*^2^ and (∂*N*/∂*Y*)^2^ contain sharp peaks. To circumvent inaccuracies caused by these peaks, Möbius transformations of the integrands were used to make the integrals more amenable to quadrature [32]. Following transformation of the integrand to smooth forms, the standard 61-point Gauss-Kronrod rule was used, as it was for Eq (A5). We then find:

$$\int_{0}^{\infty }N^{2}\left(\frac{8}{9},Y\right)dY≃0.26020.$$ (A6)

The final integral involving (∂*N*/∂*Y*)^2^ requires an additional step, i.e., calculation of the derivative ∂*N*/∂*Y*. We first used different finite-difference approximations [33]. However, the results were not reliable as *Y* → 0 so an additional Möbius transformation as used in that region, accounting for the case of *W* → 8/9, which again involves a rapidly varying integrand. The result obtained is:

$$\int_{0}^{\infty }\int_{-1}^{1}{\left(\frac{\partial N}{\partial Y}\right)}^{2}dYdr≃0.49823.$$ (A7)

## Supporting information

S1 Text Comparison of the new drag formula, Eq (10), with numerous data sets from the literature (XLSX). (XLSX)
